# Assessing sagittal discrepancies using beta angle, ANB angle and WITS appraisal: A cephalometric study

**DOI:** 10.6026/973206300214173

**Published:** 2025-11-15

**Authors:** Nakul Nandakishor Mude, Amit Kumar, Gazala Irfan, Deepti Virupakshappa, Rajashekhara Bharisharanesha, Satwik Chatterjee, Ritik Kashwani

**Affiliations:** 1 Department of Orthodontics and Dentofacial Orthopedics, ESIC Dental College and Hospital, Kalaburagi, Karnataka, India; 2 Department of Orthodontics, Muskan Dental Care, Deoghar, Jharkhand, India; 3 Department of Orthodontics & Dentofacial Orthopaedics, Hazaribag College of Dental Sciences & Hospital, Hazaribag, Jharkhand, India; 4 Department of Maxillofacial Surgery and Diagnostic Sciences, College of Dentistry, Prince Sattam Bin Abdulaziz University, Al-Kharj, Saudi Arabia; 5 Department of Pediatric Dentistry, College of Dentistry, Prince Sattam Abdul Aziz University, Kingdom of Saudi Arabia; 6 Department of Forensic Science, Adamas University, Kolkata, West Bengal, India; 7 Department of Oral Medicine and Radiology, School of Dental Sciences, Sharda University, Greater Noida, Uttar Pradesh, India

**Keywords:** ANB angle, beta angle, cephalometry, skeletal discrepancies, Wits appraisal

## Abstract

There is a need to evaluate the reliability and applicability of various cephalometric tools used for assessing sagittal discrepancies in patients with Class I, II, and III skeletal patterns. Therefore, it is of interest to assess sagittal discrepancies in patients with Class I, II, and III skeletal patterns by comparing three common diagnostic tools: the beta angle, ANB angle and Wits appraisal. Hence, a total of 120 subjects, divided into three groups based on their skeletal pattern, were evaluated using lateral cephalograms. Statistical analyses showed significant differences in beta angle measurements between the groups. The beta angle was found to provide a more consistent assessment of sagittal jaw relationships than the ANB angle and Wits appraisal, especially in cases with jaw rotations or occlusal plane changes. Thus, we show the beta angle as a valuable tool for orthodontic diagnosis, offering greater reliability in assessing skeletal relationships. Further research with larger samples and different populations is recommended to validate these findings.

## Background:

Diagnosis is a fundamental aspect of effective treatment planning, as it directly influences the approach and outcomes of medical interventions. In orthodontics, diagnosing skeletal and dental discrepancies is key to determining the most appropriate treatment strategies [1]. This process largely depends on the interpretation of various analysis methods, with particular emphasis on evaluating the sagittal apical base relationship. Both angular and linear measurements have become integral components of cephalometric analyses, assisting clinicians in diagnosing anteroposterior (AP) discrepancies and formulating the optimal treatment plan [2]. Cephalometric radiographic analysis, in particular, has been widely employed in assessing the relationships between the maxilla and the mandible, helping to pinpoint skeletal jaw discrepancies [3]. One of the most commonly used tools in this regard is the ANB angle, which represents the spatial relationship between the maxilla and mandible. However, it has been noted that the ANB angle has several limitations. Point A and Point B, key landmarks in this analysis, are dentoalveolar structures influenced by both growth and orthodontic treatment, meaning that changes in their position reflect a combination of skeletal and dental changes [4]. Additionally, the position of nasion is not fixed during growth and any displacement of nasion can directly affect the ANB angle, leading to discrepancies in interpreting skeletal relationships. While the ANB angle remains popular and useful, there are instances where it fails to fully capture the actual discrepancies between the apical bases [5]. Researchers highlighted these inadequacies, demonstrating that the ANB angle does not provide an accurate assessment of variations in skeletal relationships due to inconsistent variations in craniofacial features. These include the relative position of nasion to the jaw bones and the rotational effects of the jaw with respect to cranial reference planes [6]. To address these issues, Jacobson suggested the Wits appraisal as a more reliable alternative. The Wits appraisal offers the advantage of relating the jaw bases to cranial reference planes, thus providing a more comprehensive understanding of skeletal relationships. However, it also has limitations, especially when it comes to assessing changes in the occlusal plane rather than purely anteroposterior changes in the jaw bones [7]. To further refine the assessment of skeletal relationships, new measurements such as the beta angle have been proposed. The beta angle is independent of cranial reference planes and dental occlusion [8]. By using three skeletal landmarks-Point A, Point B and the apparent axis of the condyle-it offers a more stable and reliable method of evaluating the sagittal apical base relationship. The beta angle is particularly useful when traditional cephalometric measurements, such as the ANB angle and the Wits appraisal, are insufficient due to their reliance on variable factors [9]. Therefore, it is of interest to determine the reliability and applicability of the beta angle in assessing sagittal skeletal relationships, especially in cases where other cephalometric measures fall short.

## Methodology:

The present study was conducted in the Department of Orthodontics and Dentofacial Orthopaedics, including a total of 120 subjects. The sample consisted of three skeletal pattern groups, with 40 patients in each group. Each group was further subdivided into two sub-groups, consisting of 20 male and 20 female subjects each. The three groups were selected based on Class I, Class II and Class III skeletal discrepancies. The age limit for male patients was 18 years and above, while for female patients, the age limit was 15 years and above, ensuring standardization. Pretreatment lateral cephalograms were collected from each patient and traced individually. The breakdown of the subjects is as follows: Group I - Class I skeletal pattern (n=40) = 20 male + 20 female, Group II - Class II skeletal pattern (n=40) = 20 male + 20 female and Group III - Class III skeletal pattern (n=40) = 20 male + 20 female. The inclusion criteria for the study were as follows: for the Class I skeletal pattern group, patients had an ANB angle ranging from 2° to 4°, a Wits appraisal where AO coincided with BO or BO was ahead of AO by 1 mm and a pleasant profile. For the Class II skeletal pattern group, patients had an ANB angle greater than 4°, a Wits appraisal where BO was behind AO (positive reading) and a profile having a Class II appearance. For the Class III skeletal pattern group, patients had an ANB angle less than or equal to 2°, a Wits appraisal where BO was ahead of AO (negative reading) and a profile having a Class III appearance. The exclusion criteria included patients with craniofacial anomalies, cleft palate, or a history of previous orthodontic treatment. The materials used in this study included lateral cephalograms, matte acetate sheets, set squares, a 0.3 mm lead pencil and a tracing table. The radiology equipment used was the Sirona Orthophos XG-5 Digital Panoramic unit. For the analysis, the following parameters were measured: Beta angle, Wits appraisal and ANB angle. Beta angle was measured using the landmarks Point A (Subspinale), Point B (Supramentale) and Point C (the center of the condyle). The angle was calculated between the perpendicular line dropped from point A to the C-B line and the A-B line. Wits appraisal was assessed using Point A, Point B and the functional occlusal plane, where perpendicular lines were drawn from points A and B to the occlusal plane and the distance between these perpendiculars was measured. The ANB angle was calculated using Sella turcica (S), Point A (Subspinale) and Point B (Supramentale), by determining the difference between the SNA and SNB angles. The statistical analysis was carried out using SPSS version 20. A two-way ANOVA was performed to compare beta angle scores in the three sub-groups (I, II, III) and gender (male and female), followed by Tukey's multiple post-hoc test for pairwise comparisons. A one-way ANOVA was used to compare beta angle, Wits appraisal and ANB angle across the three sub-groups and Tukey's post-hoc test was applied for pairwise comparisons. Karl Pearson's Correlation Coefficient test was used to assess the correlation between beta angle, Wits appraisal and ANB angle in the three sub-groups. A p-value of less than 0.05 was considered statistically significant.

## Results:

The present retrospective and observational cephalometric study was planned to determine the mean values and the standard deviation for beta angle with three skeletal patterns (class I, class II and class III) in an Indian population from Marathwada region, to compare the mean values of beta angle between male and female patients; and to compare the three angles viz. beta angle, Wits appraisal and ANB angle to measure the anteroposterior dysplasia and to evaluate which one was more reliable amongst them. The study included a total of 120 subjects consisting of three skeletal pattern groups of 40 patients each that were, further, divided into two sub-groups containing 20 male and 20 female subjects. The three groups were selected based on skeletal class I, II, III patterns with age limit of 18 years and above for male and 15 years and above for female patients for standardization. Pre-treatment lateral cephalograms were collected from each patient and traced individually. The values obtained were tabulated and subjected to statistical analysis. SPSS version 20 was used for statistical analysis while the comparison in the three sub-groups (I, II, III) and gender (male and female) with respect to beta angle scores was done by two way ANOVA and pair wise comparison of the three sub-groups (I, II, III) and gender (male and female) with respect to beta angle scores was done by Tukeys multiple post-hoc test. Comparison in the three sub-groups (I, II, III) with beta angle, Wits appraisal and ANB angle was done by one way ANOVA while pair wise comparison of the three sub-groups (I, II, III) with beta angle, Wits appraisal and ANB angle was done by Tukeys multiple post-hoc test. Karl Pearson's Correlation Coefficient test was used for the correlation between beta angle, Wits appraisal and ANB angle and in the three sub-groups (I, II, III). P-values < 0.05 were considered to be statistically significant. The mean values of beta angle in the Marathwada population for the Class I skeletal pattern group were found to be 30.35° with a standard deviation of ± 2.64° for males while 31.35° with a standard deviation of ± 3.01° for females; in class II skeletal pattern group, the similar values were 21.65° with a standard deviation of ± 1.14° for males while 22.10° with a standard deviation of ± 3.09° for females; while in the class III skeletal pattern group, the values were 42.65° with a standard deviation of ± 5.13° for males while 41.10° with a standard deviation of ± 3.89° for females (Table 1 - see PDF and Figure 1 - see PDF).

Comparison in the three sub-groups (skeletal class I, II, III) with respect to beta angle scores by two ways ANOVA revealed that the 3 sub-groups were significantly different. The values were found to be statistically significant with P ≤ 0.0001 as far as the skeletal patterns were concerned. However, no significant difference could be observed with respect to gender of the patients (Table 2 - see PDF). The Tukeys multiple post-hoc test, also, found few of the sub-groups to be significantly different with statistically significant differences between the sub-groups class I and II males, class I males and III females, class I and III males, class I males and III females, class I females and II males, class I and II females, class I females and III males, class I and III females, class II and III males, class II males and III females, class II females and III males and class II and III females with P ≤ 0.0001. The corresponding values between the other sub-groups, though, were found to be statistically insignificant with P > 0.05 (Table 3 - see PDF). To summarize, the mean values of beta angle in the Marathwada population for the Class I skeletal pattern group were found to be 30.85° with a standard deviation of ± 2.84°; in class II skeletal pattern group, the similar values were 21.88° with a standard deviation of ± 2.31°; while in the class III skeletal pattern group, the values were 41.88° with a standard deviation of ± 38 4.56°. The corresponding minimum and maximum values were 26° and 35° for Class I skeletal pattern group; 16° and 28° for Class II skeletal pattern group; while 36° and 55° for Class III skeletal pattern group (Table 4 - see PDF and Figure/Graph 2). The mean values of Wits appraisal scores for Class I skeletal pattern group were found to be - 1.30° with a standard deviation of ± 1.22°; while in class II skeletal pattern group, the similar values were 5.93° with a standard deviation of ± 1.90°; and in class III skeletal pattern group, the values were -4.83° with a standard deviation of ± 0.78°. The corresponding minimum and maximum values were -3° and 2° for Class I skeletal pattern group; 2° and 10° for Class II skeletal pattern group; while -6° and -4° for Class III skeletal pattern group (Table 4 - see PDF and Figure/Graph 3). The mean values of ANB angle in the Marathwada population for Class I skeletal pattern group were found to be 2.80° with a standard deviation of ± 1.04°; while in class II skeletal pattern group, the similar values were 6.48° with a standard deviation of ± 1.41°; and in class III skeletal pattern group, the values were -5.88° with a standard deviation of ± 2.00°. The corresponding minimum and maximum values were 1° and 4° for Class I skeletal pattern group; 5° and 9° for Class II skeletal pattern group; while -9° and -2° for Class III skeletal pattern group (Table 4 - see PDF and Figure 1 - see PDF). Comparison in the three sub-groups (skeletal class I, II, III) with beta angle, Wits appraisal and ANB angle by one way ANOVA revealed statistically significant values of all the three said parameters, beta angle, Wits appraisal and ANB angle, with P ≤ 0.0001 (Table 5 - see PDF). Pair wise comparison of the three sub-groups (I, II, III) with beta angle, Wits appraisal and ANB angle by Tukeys multiple post-hoc test, also, revealed the intra-group comparisons between the three sub-groups, viz. Class I vs Class II, Class I vs Class III and Class II vs Class III to be 39 statistically significant for all the three said parameters, beta angle, Wits appraisal and ANB angle, with P ≤ 0.0001 in all the patients (Table 6 - see PDF). Correlation between beta angle, Wits appraisal and ANB angle and in the three sub-groups (I, II, III) revealed the r-value to be 0.0383 between beta angle with Wits appraisal for Class I patients, while -0.0190 for beta angle with ANB angle and 0.0723 between Wits appraisal with ANB angle for Class I patients; with the corresponding r-values of 0.1496; -0.1383; and 0.1949 for Class II; and 0.2941; 0.1112; and 0.0185 for skeletal Class III patients (Table 7 - see PDF). Overall, the corresponding correlation between beta angle with Wits appraisal, beta angle with ANB angle and Wits appraisal with ANB angle revealed the r-values to be -0.8426; -0.8722; and 0.8443 in the three sub-groups with the values obtained, also, being statistically significant with P ≤ 0.0001 (Table 7 - see PDF).

## Discussion:

Accurate anteroposterior measurement of jaw relationships is crucial in orthodontic treatment planning, as it directly influences the diagnosis and the choice of interventions. In cephalometric analysis, both angular and linear measurements are used to evaluate sagittal jaw relationships and jaw position [10]. However, angular measurements, such as the ANB angle, can be prone to errors due to factors like changes in facial height, jaw inclination and overall jaw prognathism. Similarly, linear measurements are also affected by the inclination of reference lines [11]. This variability in measurements leads to complexities in accurately interpreting jaw relationships and thus, there has been an on-going need for a more reliable method [12]. The ANB angle has long been the most popular parameter for assessing sagittal jaw relationships. However, it is influenced by several factors that can distort its interpretation. Changes in the extension and inclination of the anterior cranial base, the position of nasion, jaw rotations due to growth or orthodontic treatment and facial prognathism all affect the ANB angle, making it a less reliable indicator in certain cases [13]. In response to these limitations, the Wits appraisal was introduced as an alternative. The Wits appraisal does not depend on cranial landmarks or jaw rotations, offering a potentially more stable measure. However, identifying the occlusal plane for accurate measurement can be difficult, especially in patients with mixed dentition, open bites, or skeletal asymmetries [14]. Furthermore, changes in the Wits measurement during orthodontic treatment might reflect changes in the occlusal plane rather than the actual sagittal changes in jaw relationships [15]. To overcome these challenges, the Beta angle was introduced as a more reliable method for assessing sagittal jaw relationships. The Beta angle does not rely on cranial landmarks or the functional occlusal plane. Instead, it uses three skeletal landmarks: Point A, Point B and the condyle's apparent axis (Point C) [16]. This makes the Beta angle particularly useful in cases where other measurements like the ANB angle or Wits appraisal may be inaccurate due to jaw rotations or occlusal plane discrepancies. Unlike the ANB angle, the Beta angle remains relatively stable even when the jaws are rotated, making it more effective in assessing skeletal patterns that are subject to such rotations [17]. Researches compare the Beta angle to other cephalometric measures, such as the ANB angle and Wits appraisal, shows promising results. For instance, Qamaruddin *et al.* (2018) [18] study on the Greek population found that the Beta angle in Class I patients ranged from 27° to 35°, which aligns closely with the findings in the current study (26° to 35° for Class I). Similarly, in Class II and Class III patients, the Beta angle showed expected trends, with Class II patients exhibiting smaller angles and Class III patients' larger angles, consistent with previous research. In comparison to the ANB angle and Wits appraisal, the Beta angle demonstrated greater consistency across skeletal groups, making it a more reliable measurement [19]. Despite its advantages, the Beta angle does have limitations. Accurately locating the condyle's center can be challenging and cephalometric x-rays need to be of high quality to use the Beta angle effectively [20]. Nonetheless, its ability to reflect true sagittal changes in jaw relationships, especially throughout orthodontic treatment, makes it a valuable tool for clinicians. This study supports the Beta angle's reliability and applicability in orthodontic treatment planning, particularly when used in conjunction with other measurements to ensure a comprehensive evaluation of the patient's jaw relationship.

## Conclusion:

Beta angle provides a more reliable and consistent measurement of sagittal jaw relationships compared to the ANB angle and Wits appraisal, especially when jaw rotations or occlusal plane changes are present. Its stability and accuracy make it a valuable tool for orthodontic diagnosis and treatment planning. Therefore, the Beta angle can enhance the precision of cephalometric evaluations.
